# Melatonin enhances sorafenib actions in human hepatocarcinoma cells by inhibiting mTORC1/p70S6K/HIF-1α and hypoxia-mediated mitophagy

**DOI:** 10.18632/oncotarget.20592

**Published:** 2017-08-24

**Authors:** Néstor Prieto-Domínguez, Carolina Méndez-Blanco, Sara Carbajo-Pescador, Flavia Fondevila, Andrés García-Palomo, Javier González-Gallego, José L. Mauriz

**Affiliations:** ^1^ Institute of Biomedicine (IBIOMED), University of León, León, Spain; ^2^ Centro de Investigación Biomédica en Red de Enfermedades Hepáticas y Digestivas (CIBERehd), Instituto de Salud Carlos III, Madrid, Spain; ^3^ Service of Oncology, Complejo Asistencial Universitario de León, León, Spain

**Keywords:** hepatocarcinoma, hypoxia-inducible factor-1α, hypoxia-mediated mitophagy, melatonin, sorafenib

## Abstract

The antiangiogenic effects of sustained sorafenib treatment in hepatocellular carcinoma (HCC) lead to the occurrence of hypoxia-mediated drug resistance resulting in low therapy efficiency and negative outcomes. Combined treatments with coadjuvant compounds to target the hypoxia-inducible factor-1α (HIF-1α) represent a promising therapeutic approach through which sorafenib efficiency may be improved. Melatonin is a well-documented oncostatic agent against different cancer types. Here, we evaluated whether melatonin could enhance sorafenib cytotoxicity and overcome the hypoxia-mediated resistance mechanisms in HCC. The pharmacological melatonin concentration (2 mM) potentiated the oncostatic effects of sorafenib (5 μM) on Hep3B cells even under hypoxia. Melatonin downregulated the HIF-1α protein synthesis through the inhibition of the mammalian target of rapamycin complex 1 (mTORC1)/ribosomal protein S6 kinase beta-1 (p70S6K)/ribosomal protein S6 (RP-S6) pathway, although the indole enhanced Akt phosphorylation by the mTORC1/C2 negative feedback. Furthermore, melatonin and sorafenib coadministration reduced the HIF-1α-mitophagy targets expression, impaired autophagosome formation and subsequent mitochondria and lysosomes colocalization. Together, our results indicate that melatonin improves the Hep3B sensitivity to sorafenib, preventing HIF-1α synthesis to block the cytoprotective mitophagy induced by the hypoxic microenvironment, an important element of the multifactorial mechanisms responsible for the chemotherapy failure.

## INTRODUCTION

Hepatocellular carcinoma (HCC) represents the sixth most diagnosed cancer with rising incidence worldwide [1]. Unfortunately, most of the HCC cases are diagnosed in advanced stages, where surgery is not available, and the multikinase inhibitor sorafenib (BAY 43-9006, Nexavar^®^) is the only approved drug for palliative HCC treatments [1]. However, sustained treatments lead to drug resistance and the median overall survival is quite low [2]. Sorafenib targets the cell surface tyrosine kinase receptors vascular endothelial growth factor receptors (VEGFRs), the platelet-derived growth factor receptor-β (PDGFR-β) and their downstream kinases (Raf-1, B-Raf) to exert its antitumor effects [3].

While sorafenib moderates HCC progression in early stages, due to its antiangiogenic, antiproliferative and proapoptotic properties, continued sorafenib treatment decreases microvessel density and increases tumor hypoxia [2], leading to an imbalance between cell survival and death programs (autophagy, including mitophagy which is an autophagy form that controls mitochondrial homeostasis, and apoptosis) and contributing eventually in decreased drug sensitivity [4]. Therefore, there is an urgent need to understand the mechanisms of acquired resistance to sorafenib which include, among others, changes not only in the tumor microenvironment but also in survival and death processes, using hypoxia-related models to explore alternative compounds to improve the conventional therapy sensitivity.

Hypoxia-inducible factor-1 (HIF-1), the key regulator of glucose and oxygen homeostasis, modulates the expression of angiogenesis, metastasis and chemoresistance-related proteins at transcriptional level [5], as well as the mitophagy targets B-cell lymphoma-2 (BCL2)/adenovirus E1B 19 kDa-interacting protein 3 (BNIP3) and BNIP3-like protein X (NIX) [6]. Whereas hypoxia induces HIF-1α transcriptional activity, under normoxia HIF-1α is hydroxylated by a family of O_2_-dependent prolyl hydroxylases (PHDs), allowing its recognition by the von Hippel–Lindau protein (VHL) for ubiquitin-dependent proteasomal degradation [5]. Additionally, the phosphatidylinositol-3-kinase (PI3K)/Akt/mammalian target of rapamycin (mTOR) pathway regulates HIF-1α synthesis [7]. Sorafenib-resistant HCC patients have shown a stronger HIF-1α expression than the sorafenib-sensitive subjects [2]; hence, this factor is closely related to the acquisition of sorafenib resistance [8].

Melatonin (N-acetyl-5-methoxytryptamine), an indoleamine with antioxidant and anti-inflammatory properties [9], has been highlighted by its antitumor effects in cancer studies [10, 11] and we have described its antitumor features using *in vitro* and *in vivo* HCC models [12-18]. Recently, we reported that melatonin induces prodeath mitophagy to enhance HCC cells sensitivity to sorafenib under normoxia [19]. Subsequently, another group confirmed the melatonin ability to improve sorafenib cytotoxicity in normoxic conditions [20]. However, whether this combination could be a promising strategy to overcome hypoxia-acquired sorafenib resistance remains unknown. Thus, we decided to analyze the melatonin capacity to sensitize HCC cells to sorafenib under hypoxia, focusing on HIF-1α and the mitophagy-related pathways.

## RESULTS

### Melatonin enhances sorafenib cytotoxicity under normoxic and hypoxic conditions

Sustained sorafenib treatments for HCC are ineffective since its antiangiogenic effects lead to a selection of highly-resistant cells adapted to oxygen and nutrient deprivation [2]. Considering the previously mentioned melatonin features in HCC, we questioned whether its combination with sorafenib could effectively potentiate the sorafenib antitumor activity. We first evaluated Hep3B viability 48 h after treatment with melatonin (1 and 2 mM) and/or sorafenib (2.5, 5 and 10 μM) under normoxia or hypoxia by 3-(4,5-dimethylthiazol-2-yl)-2,5-diphenyl-tertazolium bromide (MTT) assay.

**Figure 1:**
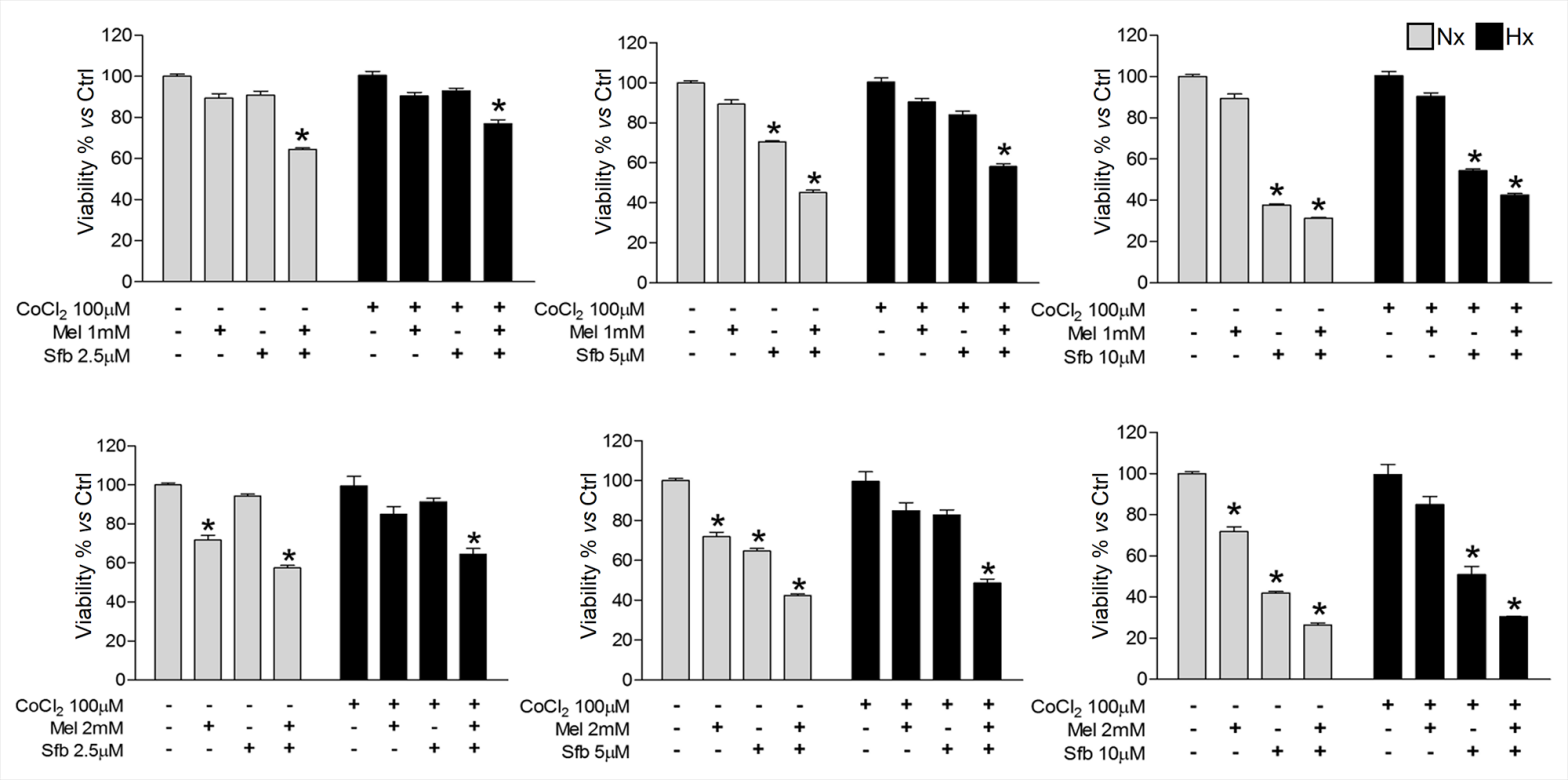
Dose-dependent Hep3B cells viability response to sorafenib and melatonin Cells were incubated under normoxia (Nx) or hypoxia (Hx) for 48 h in the absence or presence of sorafenib (2.5, 5 or 10 μM) and/or melatonin (1 or 2 mM). Viability was analyzed by MTT. Data are expressed as a percentage of mean values ± SD of experiments performed in triplicate. *p<0.05 *vs* normoxic or hypoxic non-treated cells.

When administered alone, 5 μM sorafenib and 2 mM melatonin were the minimum doses found to significantly reduce Hep3B viability, but only under normoxia, while 10 μM sorafenib was required to exert comparative effects under hypoxia. Interestingly, administration of 2 mM melatonin with all the sorafenib concentrations tested synergistically enhanced sorafenib cytotoxicity in Hep3B (Figure 1). Therefore, 2 mM melatonin and 5 μM sorafenib were selected to carry out further experiments.

### Sorafenib and melatonin coadministration reduces HIF-1α expression and Hep3B cells viability

**Figure 2:**
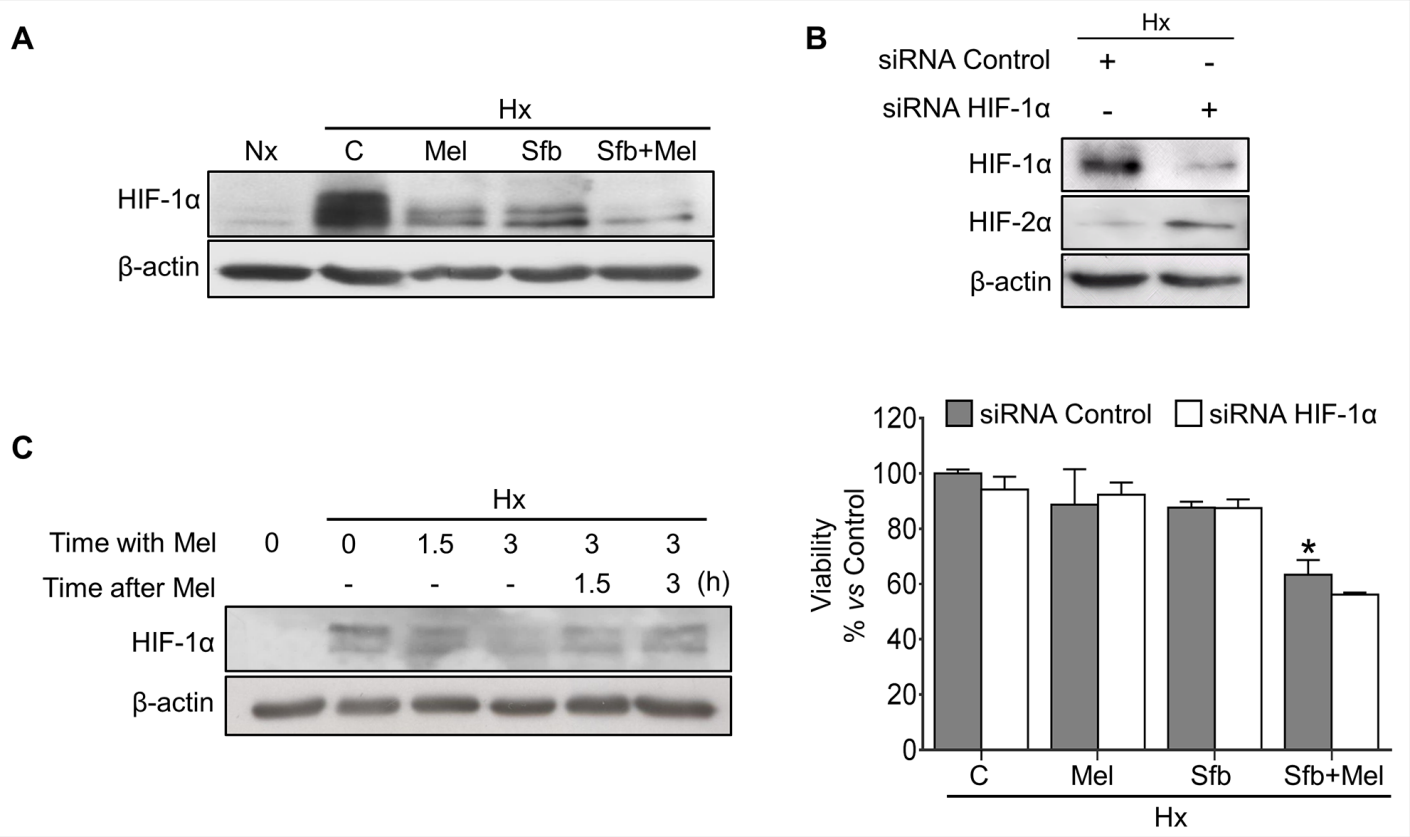
Effect of melatonin and sorafenib on HIF-1α expression and role of HIF-1α in the hypoxia-mediated resistance Hep3B cells incubated under normoxia (Nx) or hypoxia (Hx) were treated with sorafenib (5 μM) and/or melatonin (2 mM). **(A)** HIF-1α protein levels were measured by Western blot 24 h post-treatment. **(B)** Representative immunoblots of HIF-1α and HIF-2α 48 h post-treatment (upper panel). Viability was analyzed by MTT assays 48 h after the different treatments (lower panel). Data are expressed as a percentage of mean values ± SD of experiments performed in triplicate. *p<0.05 *vs* control siRNA hypoxic cells. **(C)** Representative immunoblots from the melatonin effects on HIF-1α dynamics. Hep3B cells were preincubated under hypoxia for 3 h (Lane 2), and melatonin was subsequently added for 1.5 and 3 h (Lanes 3 and 4). After melatonin removal, cells were incubated again under hypoxia conditions for additional 1.5 and 3 h (Lanes 5 and 6) to restore the initial hypoxic microenvironment. Lane 1 shows normoxic HIF-1α basal levels.

High HIF-1α levels strongly correlate with cancer progression and hypoxia-mediated sorafenib resistance [21]. The combination of sorafenib with some sort of “sensitizer” able to target HIF-1α seems a promising therapeutic approach [2]. We analyzed the effect of melatonin and/or sorafenib treatments on HIF-1α expression. As expected, hypoxic cells exhibited a robust HIF-1α accumulation (∼50 fold). When administered individually for 24 h, both drugs decreased the steady-state levels of HIF-1α protein, being melatonin more effective than sorafenib (∼4 and 1.5 fold decrease respectively *vs* hypoxic Hep3B cells). Interestingly, coadministration drastically diminished HIF-1α protein expression (∼20 fold decrease *vs* hypoxic Hep3B cells) (Figure 2A). To assess the HIF-1α involvement on the melatonin/sorafenib-induced Hep3B cell death under hypoxia, we used control and HIF-1α small interfering RNAs (siRNAs). We got ∼90% HIF-1α silencing efficiency 48 h post-treatment (Figure 2B). Although we detected a slight tendency suggesting that the silencing and the dual treatment might exert synergic effects, we did not observe statistically significant differences in Hep3B viability between control and HIF-1α siRNAs cells after treatments. These results could be explained by a compensatory mechanism by which HIF-2α is upregulated when the HIF-1α expression is reduced (Figure 2B) [22]. To explore the melatonin-mediated dynamic changes on HIF-1α expression, cells were preincubated under hypoxia for 3 h and subsequently exposed to the hormone for additional 3 h within the hypoxia treatment. Finally, melatonin was withdrawn for the last 3 h to restore the initial hypoxia conditions. Melatonin decreased HIF-1α protein levels in a time-dependent manner, whereas after melatonin removal, maintaining hypoxia, the HIF-1α levels were progressively restored (Figure 2C).

### Melatonin does not affect HIF-1α transcription or degradation but decreases HIF-1α protein synthesis

**Figure 3:**
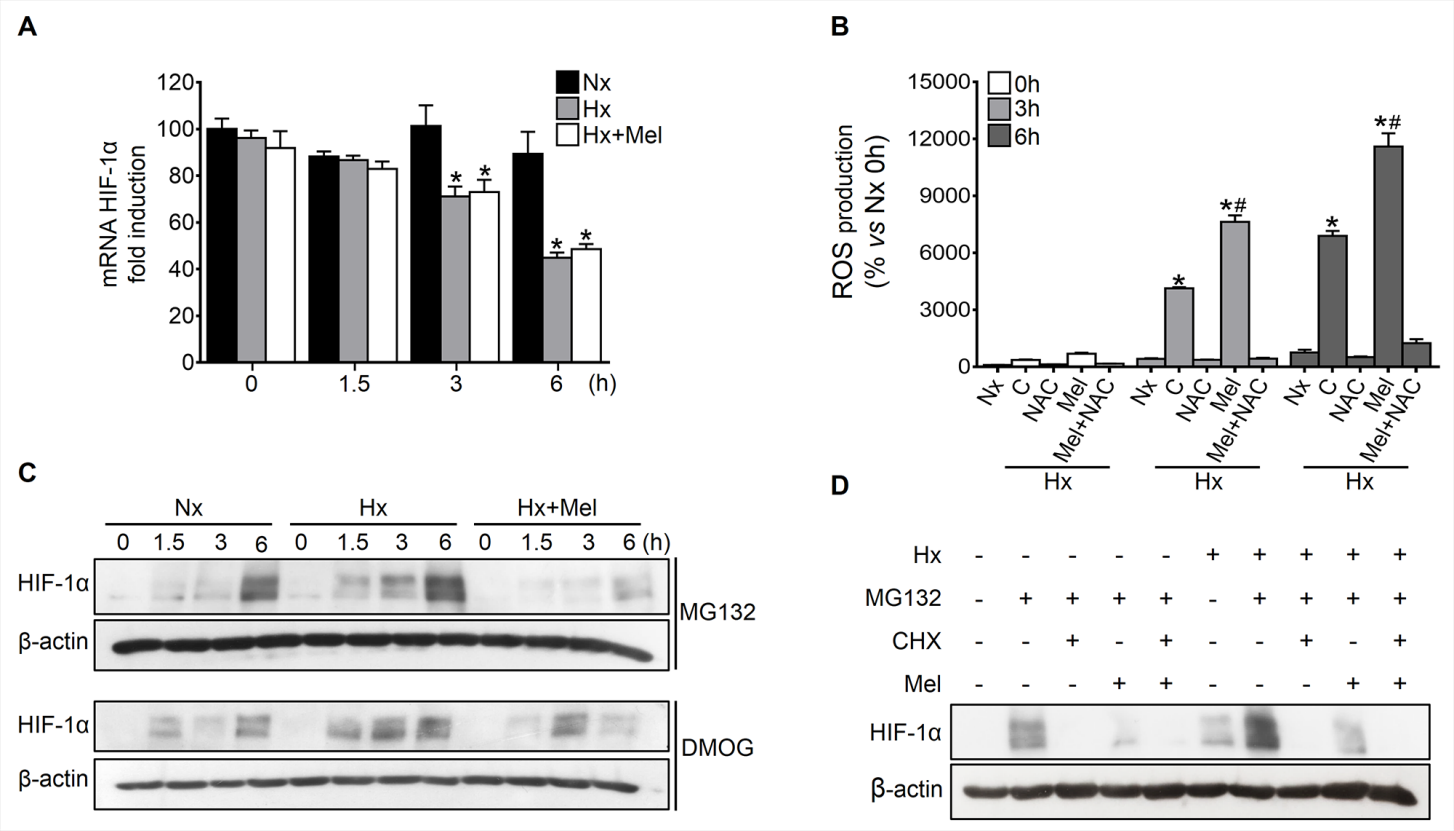
Effect of melatonin on HIF-1α transcription, protein synthesis and degradation Hep3B were incubated under normoxia (Nx) or hypoxia (Hx) in the absence or presence of melatonin (2 mM). **(A)** HIF-1α mRNA levels were measured by qRT-PCR at 0, 1.5, 3 and 6 h after treatment. Data are expressed as mean values of arbitrary units (a.u.) ± SD of three independent experiments. *p<0.05 *vs* normoxia at the same time. **(B)** ROS production was measured using DCF quantification under normoxia and hypoxia plus melatonin and/or NAC (5 mM) treatment for 0, 3 and 6 h. Data are expressed as a percentage of mean values ± SD of experiments performed in triplicate. *p<0.05 *vs* normoxia at the same time, #p<0.05 significant differences between melatonin-treated *vs* untreated cells under hypoxia. **(C)** Effect of melatonin on HIF-1α synthesis was assayed by Western blot. Cells were incubated under normoxia or hypoxia for 0, 1.5, 3 and 6 h in the absence or presence of MG132 (10 μM), DMOG (1 mM) or melatonin. **(D)** Effect of melatonin on HIF-1α degradation analyzed by Western blot. Cells were incubated under normoxia or hypoxia for 6 h with or without MG132 (10 μM), CHX (100 μM) or melatonin.

The melatonin-mediated changes on the steady-state HIF-1α expression levels could account for the melatonin ability to modulate its transcription and/or the rate between protein synthesis and degradation [23, 24]. HIF-1α mRNA levels remained unchanged under normoxia, whereas progressively decreased upon prolonged hypoxia induction; moreover, melatonin administration to hypoxic cells did not induce significant changes on HIF-1α mRNA levels (Figure 3A), suggesting that melatonin downregulates HIF-1α expression at a post-transcriptional level.

Oxygen is a required cofactor for PHDs-mediated HIF-1α proteasomal degradation and, not surprisingly, reactive oxygen species (ROS) appear to negatively regulate PHDs activity [25]. Melatonin is a well-documented antioxidant with prooxidant actions able to promote ROS generation at pharmacological concentrations (μM to mM range) in several tumor cells [10]. Since ROS production induced by melatonin seems to be context specific, mainly in tumor cells, this indole is considered to be a conditional prooxidant [26]. Both hypoxia and melatonin seemed to enhance ROS levels similarly (Figure 3B), leading us to consider very unlikely that the melatonin-dependent HIF-1α levels reduction might be a consequence of the PHDs reactivation.

To reveal the melatonin effects on HIF-1α protein synthesis, we blocked HIF-1α protein degradation with the proteasome inhibitor MG132 or the PHDs specific repressor DMOG. The blockage of both degradation processes resulted in a pronounced and progressive accumulation of HIF-1α protein. Contrariwise, HIF-1α accumulation was significantly lower in melatonin-treated hypoxic cells (Figure 3C). Moreover, the inhibition of protein synthesis by cycloheximide (CHX) almost completely prevented HIF-1α accumulation in the presence of MG132 (Figure 3D). Similarly, the addition of melatonin resulted in a substantial reduction of HIF-1α, both under normoxia and hypoxia (Figure 3D), suggesting the melatonin capacity to interfere with HIF-1α protein synthesis.

### Melatonin downregulates the mTOR complex 1 (mTORC1)/ribosomal protein S6 kinase beta-1 (p70S6K) pathway to inhibit HIF-1α protein synthesis

**Figure 4:**
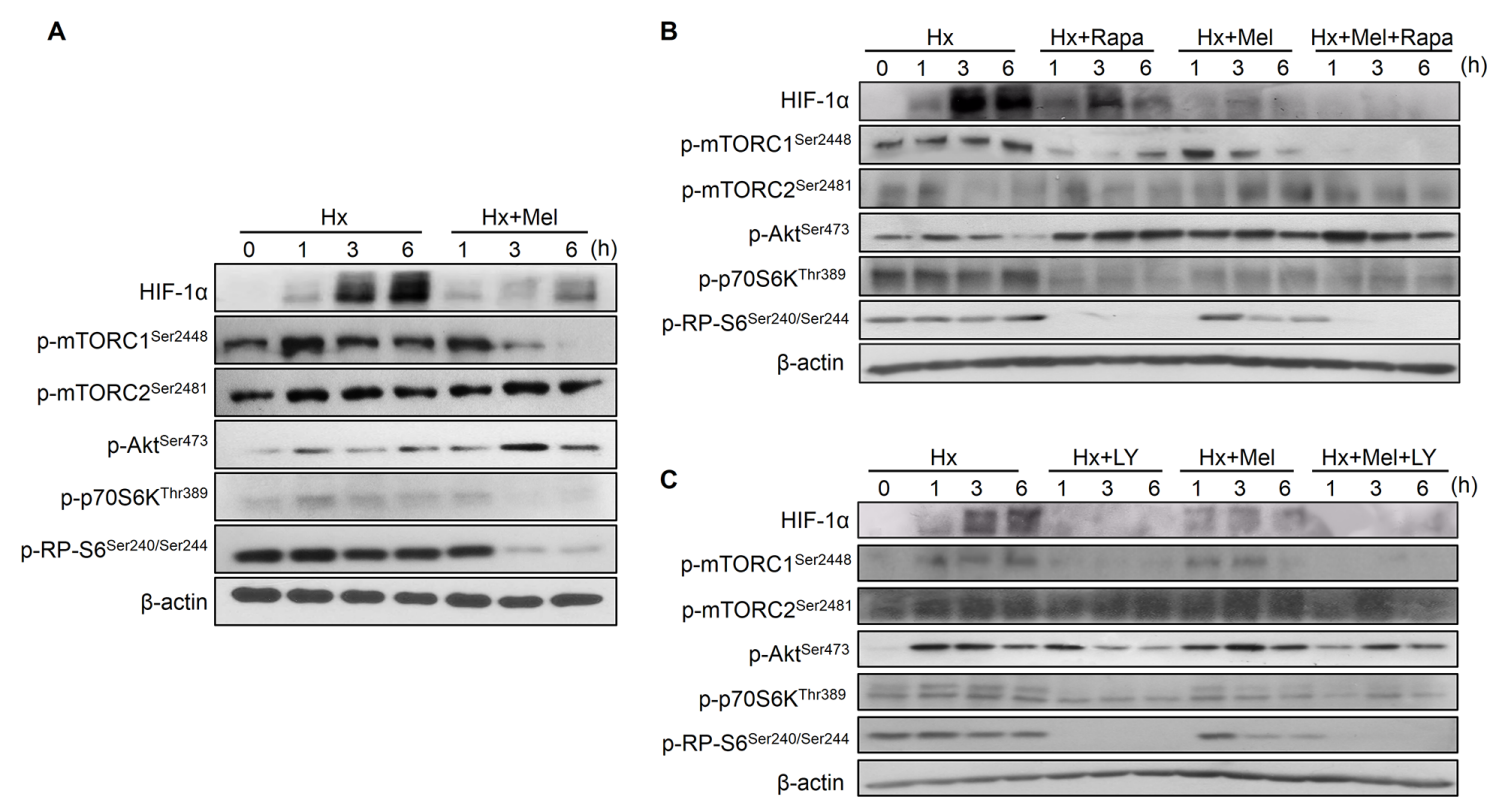
Impact of melatonin on PI3K/Akt/mTOR, HIF-1α synthesis pathway assayed by Western blot **(A)** Cells were incubated under hypoxia (Hx) for 0, 1, 3 and 6 h with or without melatonin (2 mM). Lane 1 of each panel shows normoxic basal protein levels. **(B)** and **(C)** Effect of rapamycin (20 nM) and LY294002 (50 μM) alone or in combination with melatonin on HIF-1α and PI3K/Akt/mTOR-related proteins measured by Western blot.

The PI3K/Akt/mTOR pathway regulates HIF-1α at translational levels [27]. We found that melatonin significantly decreased the phosphorylation/activation state of mTORC1, its downstream kinase p70S6K, and its effector ribosomal protein S6 (RP-S6) in a time-dependent manner, which correlated with the observed HIF-1α reduction under hypoxia. Besides, Hep3B showed an increase of mTOR complex 2 (mTORC2) and Akt phosphorylation after melatonin plus hypoxia treatment (Figure 4A).

Rapamycin addition reduced mTORC1, p70S6K, RP-S6 phosphorylation and accordingly HIF-1α synthesis, although to a lesser extent than melatonin. Similarly, mTORC2 and Akt phosphorylation levels were also increased by rapamycin (Figure 4B). Otherwise, LY294002 downregulated Akt, mTORC1, mTORC2, p70S6K and RP-S6 phosphorylation both alone and in combination with the indole, and reduced HIF-1α accumulation more sharply than melatonin (Figure 4C).

### Melatonin increases sensitivity to sorafenib through inhibition of hypoxia-induced mitophagy

**Figure 5:**
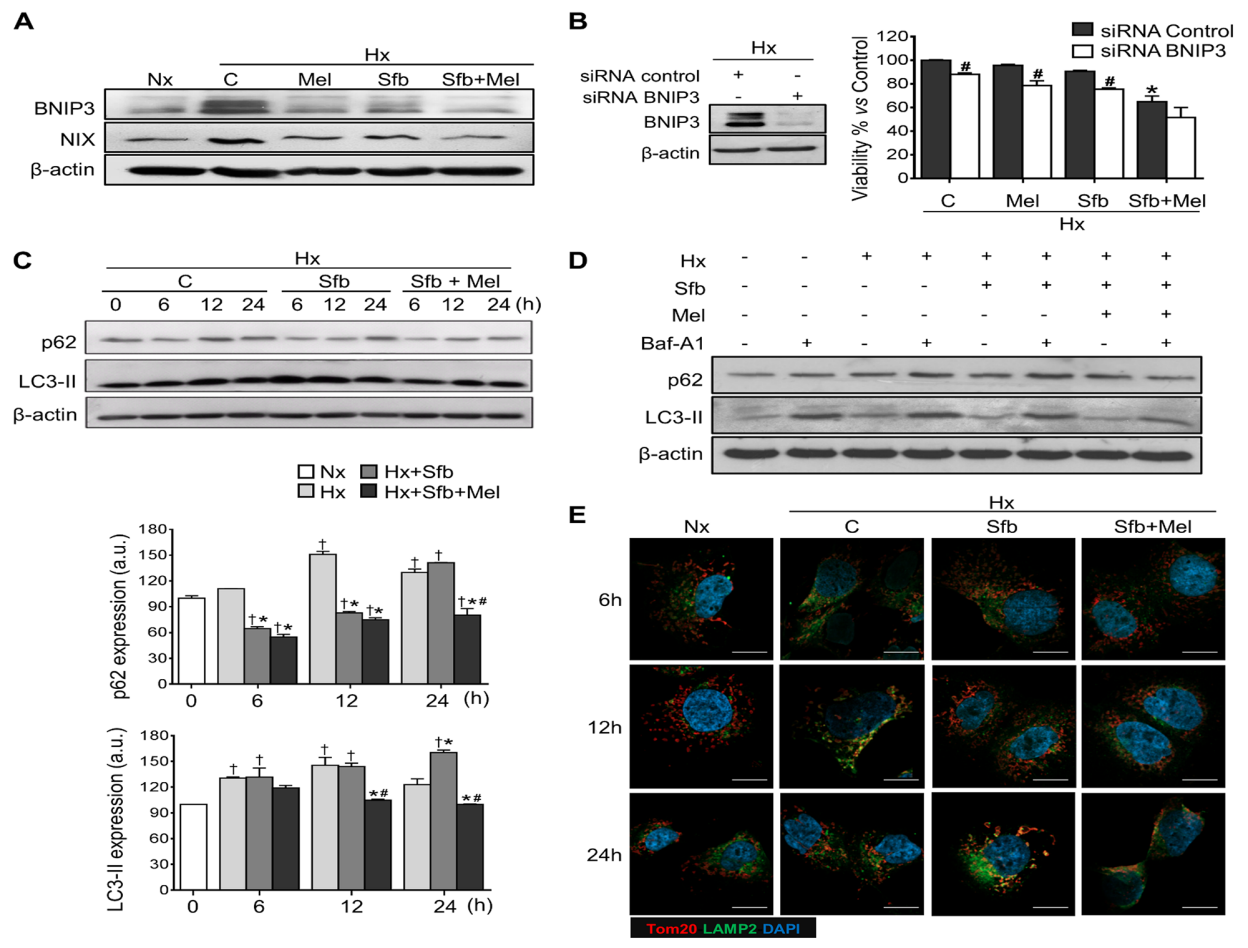
Effect of melatonin and sorafenib on hypoxia-induced mitophagy and role of mitophagy in sorafenib hypoxia-mediated resistance Hep3B cells were incubated under normoxia (Nx) and hypoxia (Hx) in the absence or presence of sorafenib (5 μM) and/or melatonin (2 mM). **(A)** BNIP3 and NIX levels were analyzed by Western blot 24 h post-treatment. Lane 1 shows basal protein levels under normoxia. **(B)** Cell viability from non-silenced and silenced cells was analyzed by MTT assay 48 h post-treatment. Data are expressed as a percentage of mean values ± SD of experiments performed in triplicate. *p<0.05 *vs* control siRNA hypoxic cells, #p<0.05 significant differences between BNIP3 and control siRNAs cells under the different treatments. **(C)** p62 and LC3-II were analyzed by Western blot 6, 12 and 24 h after treatment. Lane 1 of each panel shows its normoxic basal levels. Immunoblots were quantified using ImageJ software. Data are expressed as mean values of arbitrary units (a.u.) ± SD of three independent experiments. †p<0.05 *vs* normoxia, *p<0.05 *vs* hypoxia, #p<0.05 *vs* significant differences between cotreated vs sorafenib-treated cells under hypoxia at the same time. **(D)** Immunoblots of p62 and LC3-II from Hep3B cells incubated with the indicated treatments for 12 h and with or without Baf-A1 (5 μM) for the last 3 h. **(E)** Confocal images show mitochondria and lysosomes localization in Hep3B up to 24 h, using Tom20 (red) and LAMP2 (green) antibodies respectively. DAPI (blue) denotes cell nucleus. Magnification is 63X and scale bar represents 15 μm.

Mitophagy, a specific form of autophagy, controls mitochondrial homeostasis against different cellular stresses like nutrient or oxygen depletion [6]; thus, targeting mitochondria for therapeutic purposes represents a very attractive approach. We evaluated the sorafenib and/or melatonin effects on hypoxia-mediated mitophagy in Hep3B. Hypoxia strongly induced the expression of BNIP3 and NIX (Figure 5A), mitophagy mediators directly regulated by HIF-1α [6]. The melatonin and sorafenib single treatments diminished their expression by 1-3 fold, and cotreated cells showed even lower levels that were equivalent to those found in normoxia (Figure 5A).

Mitophagy exerts a dual function in cancer, promoting cell survival or death depending on the cellular context and tumor microenvironment [28]. To elucidate the role of the hypoxia-induced mitophagy in our *in vitro* HCC model, we assessed the impact of BNIP3 gene-silencing on cell viability after melatonin and sorafenib treatments. BNIP3 expression was almost completely abolished 48 h post-treatment (Figure 5B). BNIP3 depletion resulted in increased melatonin and sorafenib sensitivity and reduced cell viability (Figure 5B), suggesting a cytoprotective role of hypoxia-related mitophagy in HCC cancer cells.

Hypoxia increased the autophagosome markers, sequestosome-1 (p62) and LC3-II (lipidated form of microtubule-associated protein I/II-light chain 3 (LC3)), expression over time, reaching a maximum peak at 12 h after treatment, which declined thereafter. Sorafenib delayed this peak until 24 h and the melatonin coadministration reduced p62 and LC3-II levels to those observed in normoxic cells (Figure 5C).

Complementary, we evaluated the functional impact of melatonin on mitophagy using 5 μM bafilomycin A1 (Baf-A1), an inhibitor of autophagosome-lysosome fusion. As shown in Figure 5D, when this process was blocked, both p62 and LC3-II strongly accumulate. While single administration of sorafenib did not significantly alter this phenotype, its combination with melatonin significantly reduced p62 and LC3-II levels, even in the presence of Baf-A1.

12 h of oxygen deprivation induced organelle fusion as indicated by colocalization between the mitochondrial marker outer mitochondrial membrane receptor (Tom20) and the lysosomal marker lysosome-associated membrane glycoprotein 2 (LAMP2). While sorafenib delayed their interaction until 24 h, the addition of melatonin with sorafenib completely abolished it (Figure 5E). Summarizing, these results endorse the melatonin capacity to impair hypoxia-mediated autophagosome formation and subsequent mitophagy in HCC cells.

## DISCUSSION

Sorafenib resistance is a relevant limitation for the HCC treatment and constitutes a multifactorial problem which has an important complexity. Although it has been clearly established that an important factor accounting for chemoresistance is the reduced ability of sorafenib to reach the site of action inside the tumor cells by reduced expression/function of its main carriers, such as OCT1, a number of additional mechanisms could be also implicated [29, 30]. While genetic heterogeneity of HCC cells could account for the primary resistance to sorafenib, its multikinase inhibitor actions promote the crosstalk between several signaling pathways, leading to the appearance of acquired resistance and consequent ineffectiveness [31, 32].

Mechanisms of chemoresistance (MOC) have been previously classified into five different groups depending on changes in drug uptake or enhanced drug export (MOC-1), poor intracellular prodrugs activation or increase on drug inactivation (MOC-2), changes in molecular targets (MOC-3), tumor cell ability to repair drug-induced changes in DNA (MOC-4) and changes in the balance between antiapoptotic and proapoptotic factors leading to cell survival (MOC-5) [33]. Furthermore, two additional MOC have been added more recently: chemoresistance due to changes in the tumor microenvironment, including hypoxia induction, (MOC-6) and chemoresistance due to phenotype transition of tumor cells (MOC-7) [34]. Cancer cells develop complex compensatory survival mechanisms to grow under hypoxia, avoid apoptosis, migrate and eventually, sustain the tumor progression even in the presence of the chemotherapy [31]. This reinforces the urgent need to elucidate the cellular mechanisms underlying the acquired resistance to sorafenib to develop more effective therapies.

The combination of sorafenib with other molecules to promote its therapeutic effects, overcome drug-resistance and mitigate side effects appears as an attractive strategy against HCC [31]. To date, the sorafenib combined therapies include anticancer drugs like arsenic trioxide [35], the member of the statins group, fluvastatin [36], the cyclooxygenase-2 inhibitor, celecoxib [37], or natural compounds such as silibinin [38] and curcumin [39], that have been reported to enhance *in vitro* cytotoxicity in HCC. Unfortunately, a perfect compound has not been found yet.

The antiproliferative, proapoptotic, antiangiogenic and antimetastatic effects of melatonin in HCC have been well-documented [12-18]. Moreover, this indole has been used as an effective coadjuvant in other therapies, overcoming clofarabine resistance in NALM6/P and SKW3/P leukemic cell lines [40], supporting doxorubicin effects in MCF-7 breast cancer cells [41] and synergizing the chemotherapeutic effects of 5-fluorouracil in SW620 and LOVO colon cancer cells [42]. However, physiological doses of melatonin did not carry on its oncostatic and antiangiogenic effects in cultured HCC cells, thus pharmacological dosages should be used [14]. Consequently, we aimed to evaluate whether the coadministration of melatonin and sorafenib could improve its cytotoxicity in HCC cells, and potentially impact the mechanisms implicated in sorafenib resistance.

The combination of pharmacological concen-trations of melatonin (1 and 2 mM) with sorafenib (2.5, 5 and 10 μM) significantly enhanced the sorafenib cytotoxicity in Hep3B cells under normoxia. Sorafenib decreases HCC vasculature, although sustained treatment increases intratumor hypoxia and associated chemoresistance [2]. Surprisingly, only a few studies have taken into consideration the implication of the hypoxic microenvironment in the HCC resistance to sorafenib in their experimental designs [8]. In order to resemble the tumor microenvironment more accurately, our experiments were performed using CoCl_2_ to mimic hypoxia. Under these conditions, melatonin also enhanced the sorafenib cytostatic effects. Moreover, the use of melatonin allowed us to lower the sorafenib dose (from the 10 μM one, commonly used in *in vitro* studies, to 5 μM). Thus, these data suggest that a combined melatonin-sorafenib therapy when hypoxia is established would be effective to potentiate the sorafenib effects, and would have great cost-benefit advantages considering the high cost of the current sorafenib-based therapies [43].

HIF-1 overexpression correlated with poor prognosis and chemoresistance [21]. HIF-1α levels are higher in sorafenib-resistant HCC tissue samples than in the sorafenib-sensitive ones [2] since, through HIF-1, resistant cells express genes implicated in proliferation, angiogenesis, migration, and autophagy to counteract hypoxia and survive [5]. Hence, targeting HIF-1α represents an interesting approach for HCC management [21]. Accordingly, EF24, a curcumin analog, overcomes sorafenib resistance promoting the VHL-dependent HIF-1α degradation [2], the histone deacetylase inhibitor vorinostat has been shown to inhibit HIF-1α translation through the eukaryotic translation initiation factor 3 subunit G (eIF3G) repression [44], and several miRNAs, such as miR-338-3p and miR-199a-5p, sensitize HCC models to sorafenib by targeting HIF-1α [45, 46].

Melatonin reduces HIF-1α levels in several cancer cell lines [25], although the underlying mechanisms remain unclear. Here, sorafenib and melatonin alone diminished the hypoxia-mediated HIF-1α accumulation, however melatonin addition contributed to a higher sorafenib-HIF-1α reduction. Likewise, cotreatment and HIF-1α silencing exhibited synergic cytotoxicity on Hep3B cells, although we did not find significant differences between silenced and non-silenced cells after treatments. HIF-1α knockdown increased HIF-2α expression to compensate HIF-1α downregulation. This isoform is also involved in tumor promotion, but its expression is restricted to certain cell types [22, 27]. While HIF-1α responses to acute hypoxia, HIF-2α is associated with chronic situations. Apparently, the sorafenib-mediated HIF-1α inhibition might switch the hypoxic response to some HIF-2α-dependent pathways [22]. Nonetheless, here we focused on the study of the mechanisms underlying the melatonin-mediated HIF-1α regulation, leaving the HIF-2α implication in HCC drug resistance for future studies.

HIF-1α levels are tightly regulated [24]; thus, the observed diminution in the HIF-1α steady-state levels after both drug coadministration could be explained by the melatonin ability to modulate either HIF-1α transcription, synthesis, or degradation processes. Our results revealed that melatonin diminished HIF-1α protein synthesis rate without affecting its PHDs-dependent degradation nor its transcription. Similarly, melatonin was found to downregulate HIF-1α synthesis in DU145 prostate cancer cells [24]. Evidence suggests that the mechanism underlying HIF-1α melatonin-dependent regulation might be cell type specific; hence, in U251 and U87 glioblastoma cells [47], and in HCT116 colon cancer cells [23], melatonin prevents ROS-dependent PHDs inactivation, promoting HIF-1α proteasomal degradation under hypoxia [25]. In our situation, melatonin behaved as prooxidant and therefore, as ROS-dependent PHDs inactivator, being unlikely that melatonin mediates the HIF-1α degradation. Although mechanisms implicated in ROS induction by melatonin remain still unclear, it has been indicated that they could be mediated by calmodulin or its ability to interact with mitochondrial electron transport chain complex III [26].

Growth factors, oncoproteins and cytokines can regulate HIF-1α translation *via* PI3K/Akt/mTOR pathway [3]. mTORC1 controls proliferation driving the ribosomal biogenesis, phosphorylating p70S6K to activate RP-S6, and the eukaryotic translation initiation factor 4E-binding protein 1 (4E-BP1). Otherwise, although Akt is mainly regulated by PI3K, mTORC2 also phosphorylates Akt. This mechanism depends on the mTORC1 effector, p70S6K, which can repress the PI3K/Akt pathway through mTORC2 inhibition [3, 27].

Melatonin prevented mTORC1, p70S6K and RP-S6 phosphorylation to reduce HIF-1α synthesis. Similar results were reported in prostate cancer cells [24]. Analogously, the flavonoid silibinin targets this pathway to inhibit HIF-1α synthesis in Hep3B and HeLa cells [7]. Furthermore, melatonin induced mTORC2 and Akt phosphorylation by mTORC1 downregulation, a negative feedback that has been previously described in HCC by similar compounds [7].

Rapamycin is a mTORC1 universal inhibitor, but a cell-type inhibitor of mTORC2 [7]. In our model, rapamycin-dependent mTORC1 downregulation enhanced mTORC2/Akt phosphorylation. Likewise, the melatonin-mediated mTORC1 reduction activated this negative loop independently of PI3K phosphorylation, since its combination with LY294002 did not completely abolish Akt activation. Melatonin can act as an Akt inductor [48] or repressor [42], while its repercussion on the mTOR pathways is unclear. Interestingly, melatonin diminished more sharply HIF-1α accumulation than rapamycin, suggesting that additional pathways other than the mTORC1/p70S6K/RP-S6 might mediate the melatonin effects, such as MAPKs [3, 27].

The HIF-1α targets, BNIP3 and NIX, are the two main mitophagy adaptors and, in the liver, BNIP3 hypoxia-induction is stronger and faster than NIX [6]. Both interact with LC3-II in the autophagosomes membrane to target damaged mitochondria for degradation [6]. Otherwise, p62 drives ubiquitinated proteins to the autophagosomes through its binding to LC3-II [28]. Successively, autophagosomes fuse with lysosomes forming autolysosomes where their content is degraded by specific lysosomal hydrolases [49].

We found a sorafenib-dependent BNIP3 and NIX reduction under hypoxia; nevertheless, this decrease only delayed the hypoxia induced-mitophagy from 12 to 24 h, as revealed by the mitochondria and lysosomes colocalization and the autophagosome markers p62 and LC3-II. BNIP3, NIX, p62 and LC3-II levels were considerably lower upon melatonin plus sorafenib coadministration as a consequence of a drastic HIF-1α downregulation. Besides, the combined treatment abrogated autophagosome formation and mitochondria and lysosomes colocalization.

In early phases of cancer development BNIP3 acts as a tumor suppressor, preventing ROS-related DNA damage to protect cells from malignant transformation; while in later stages, mitophagy allows tumor cells survival under stress conditions like hypoxia, contributing to chemoresistance [28]. BNIP3 gene-silencing resulted in a significant reduction of Hep3B viability, indicating that mitophagy exerts a protective role to sustain HCC cell growth under hypoxia. BNIP3 upregulation is known to induce prosurvival patterns and tumor aggressiveness in several cancers [28].

Supporting our results, the combination of mitophagy inhibitors with chemotherapy has already shown promising results; for instance, the mitophagy and mitochondrial division inhibitor (mdivi-1) plus cisplatin enhances cancer cell clearance [50] and reduces doxorubicin-related side effects [51]. The combination of doxorubicin with liensinine, a mitophagy flux inhibitor, enhances doxorubicin-related cancer cell death [52], and when sorafenib is administered together with Baf-A1 strongly reduces tumor volume and metastasis occurrence in HCC mice [53].

We previously reported that melatonin induces prodeath mitophagy in Hep3B cells when is administered plus sorafenib under normoxia [19]. However, as we previously mentioned, here we are resembling more advanced tumor stages associated with lower oxygen concentrations, where mitophagy exerts a prosurvival role [28].

**Figure 6:**
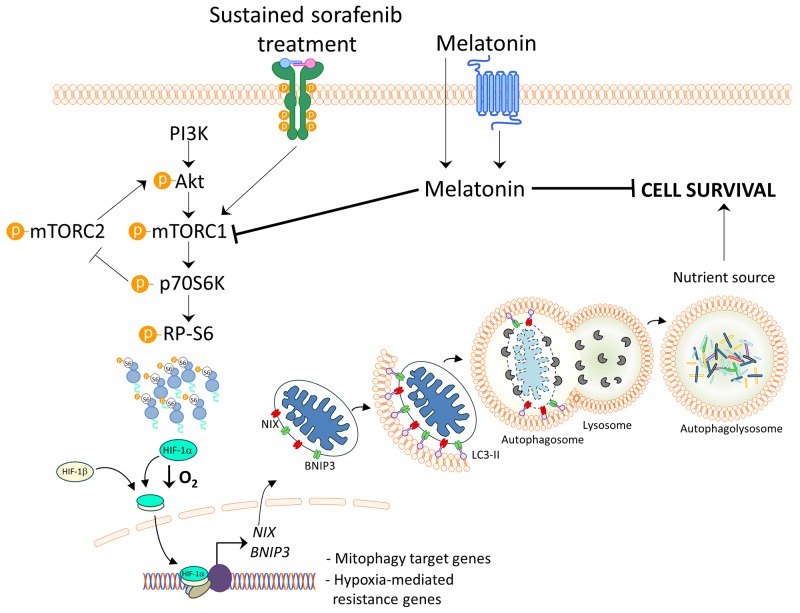
Model of melatonin inhibition of hypoxia-induced mitophagy in HCC Melatonin inhibits HIF-1α synthesis blocking subsequent hypoxia-mediated mitophagy induced by sustained sorafenib treatment. Melatonin downregulates mTORC1 and its effectors, p70S6K and RP-S6, for HIF-1α protein synthesis inhibition. Thus, HIF-1α inhibition by melatonin diminishes the transcription of the mitophagy initiators BNIP3 and NIX, preventing the prosurvival mitophagy.

It has been suggested that melatonin could promote sorafenib-induced apoptosis through c-jun N-terminal kinase (JNK)/c-jun pathway activation [20]. Nonetheless, here we proved for the first time the melatonin ability to enhance sorafenib oncostatic effects on HCC cells under hypoxia, known phenotype associated with chemotherapy failure, which has to be considered to accurately reproduce advanced HCC features *in vitro*. Moreover, we described the mTORC1/p70S6K/HIF-1α pathway inhibition and the subsequent cytoprotective mitophagy blockage as the mechanism underlying the melatonin functions, which is illustrated in Figure 6. Summarizing, the present *in vitro* study suggests that this indole could be a useful coadjuvant to overcome sorafenib resistance in advanced HCC, setting the basis for further *in vivo* studies.

## MATERIALS AND METHODS

### Cell culture

Hep3B cells from the American Type Culture Collection (Manassas, VA, USA) were grown in Dulbecco’s Modified Eagle’s medium-high glucose, supplemented with 10% fetal bovine serum, penicillin/streptomycin (100 U/ml) in a humidified 5% CO_2_ atmosphere at 37 °C. Cell culture reagents were purchased from Sigma (St Louis, MO, USA). For treatments, regular medium was replaced with fresh medium containing 1 or 2 mM melatonin (Sigma) with or without sorafenib (Santa Cruz Biotechnology, Dallas, TX, USA) at 2.5, 5 and 10 μM. Both reagents were dissolved in DMSO (Sigma) (0.2% at final concentration). CoCl_2_ (Panreac AppliChem, Barcelona, Spain) was added at 100 μM to mimic hypoxia [14]. We used 5 μM Baf-A1, 100 μM CHX, 1 mM DMOG, 50 μM LY294002, 10 μM MG132 (Tocris Bioscience, Bristol, UK) and 20 nM rapamycin (VWR, Radnor, PA, USA).

### Cell viability assay

For the MTT (Sigma) cell viability assay, 48 h after treatments media were replaced with free-serum media with MTT dissolved in PBS for 3 h at 37 °C. Next, the MTT precipitates were dissolved in DMSO and the optical densities were measured at 560 nm spectral wavelength using a microtiter plate reader (Synergy™ HT Multi-Mode Microplate Reader; BioTek Instruments, Inc., Winooski, VT, USA).

### Western blot assay

After treatments, cells were washed with ice-cold PBS and were lysed in a buffer containing 0.25 mM sucrose, 10 mM Tris and 1 mM EDTA with protease and phosphatase inhibitors (Roche Diagnostics, Basilea, Suiza) by sonication during 2 pulses of 20 s at 60% amplitude, and centrifuged for 10 min at 14,000 g. Equal amounts of protein were separated by SDS-PAGE, transferred to a PVDF membrane (Bio-Rad, Hercules, CA, USA) and were blotted with: HIF-1α (ab2185, Abcam, Cambridge, UK), HIF-2α (ab20654, Abcam), NIX (ab109414, Abcam), p-mTOR^Ser2448^ (ab109268, Abcam), BNIP3 (sc-56167, Santa Cruz Biotechnology), p-mTOR^Ser2481^ (sc-293132, Santa Cruz Biotechnology), LC3 (PM036, MBL International, Woburn, MA, USA), p62 (#5114, Cell Signaling, Beverly, MA, USA), p-RP-S6^Ser240/244^ (#5364, Cell Signaling), p-p70S6K^Thr389^ (#9206, Cell Signaling) and p-Akt^Ser473^ (#4060, Cell Signaling) antibodies, using β-actin protein (A3854, Sigma) as loading control. After three PBS-T washes, membranes were incubated for 1 h at room temperature with the antirabbit (31460, Thermo Fisher Scientific) and antimouse (P0260, Dako, Glostrup, Denmark) secondary antibodies. Proteins were visualized using Pierce ECL Western Blotting Substrate (Thermo Fisher Scientific). Specific band density was measured with ImageJ software (National Institute of Mental Health, Bethesda, MD, USA).

### Gene-silencing

Commercial siRNAs against HIF-1α (sc-35561), BNIP3 (sc-37451) and control siRNA encoding a non-targeting sequence (sc-37007) were purchased from Santa Cruz Biotechnology and introduced into Hep3B cells by reverse transfection using Lipofectamine^®^ RNAiMAX Reagent (Thermo Fisher Scientific) according to the manufacturer’s instructions. 16 h after transfection cells were treated with or without CoCl_2_, sorafenib or melatonin, and subjected to viability assay.

### Real-Time quantitative reverse transcription PCR (qRT-PCR)

After treatments, total RNA was obtained using TRIzol® Reagent (Applied Biosystems, Carlsbad, CA, USA). Residual DNA was removed using RQ1 RNase-free DNase kit (Promega, Madison, WI, USA) and successively, total RNA (500 ng) was reverse transcribed to cDNA using a High Capacity cDNA reverse transcription kit (Applied Biosystems). Next cDNA was amplified using FastStart TaqMan Probe Master (Roche) on a StepOnePlus Real-Time PCR Systems (Applied Biosystems). TaqMan primers and probes for *HIF1A* gene (NM_001243084.1 and Hs00153153_m1) and the glyceraldehyde-3-phosphate dehydrogenase (*GAPDH*) gene (NM_002046.3 and 4326317E) were derived from the commercially available TaqMan Gene Expression Assays (Applied Biosystems). Relative changes in gene expression levels were determined using the 2^-ΔΔCt^ method [54].

### ROS measurement

Treated cells were incubated with 20 μM DCF-DA (Sigma) and 15 mM Hepes (Sigma). Right after treatment, fluorescence was measured in a microplate fluorescence reader (BioTek Instruments Inc.) at 485/520 nm of excitation/emission wavelengths. N-acetyl-cysteine (NAC) (Sigma) at 5 mM was used as a ROS quencher and hydrogen peroxide (Merck, Darmstadt, Germany) as positive control. Results are expressed as the percentage of fluorescence intensity versus normoxia at 0 h.

### Immunofluorescence and laser confocal imaging

For immunofluorescence colocalization between lysosomes and mitochondria, Hep3B cells were seeded on gelatin-coated coverslips. After treatments, cells were fixed with 4% paraformaldehyde (Thermo Fisher Scientific) for 15 min, washed with PBS and permeabilized with 0.2% saponin (Sigma) in PBS and 1% fatty acid-free BSA (Sigma), all at room temperature. After washing cells with PBS, they were stained overnight at 4 °C with the [H4B4] LAMP2 (ab25631, Abcam) and the Tom20 (sc-11415, Santa Cruz Biotechnology) antibodies. Afterward, cells were washed with PBS and incubated for 1 h at room temperature with Alexa 488-conjugated antimouse (Z25002, Molecular Probes, Eugene, OR, USA) and Alexa 647-conjugated antirabbit (Z25308, Molecular Probes) IgGs. Coverslips were washed with PBS and mounted on glass slides with fluorescent mounting medium Fluoroshield™ containing 4′6-diamidino-2-phenylindole (DAPI) (Sigma) to be visualized in a Zeiss LSM 800 confocal laser scanning microscope (Zeiss AG, Jena, Germany) and samples were analyzed using ZEN software (Zeiss AG, Jena, Germany).

### Statistical analysis

Results were expressed as mean values ± SD of three independent experiments. They were analyzed by the statistical package GraphPad Prism 6 (GraphPad Software, San Diego, CA, USA), using one-way ANOVA followed by Bonferroni post-hoc test to measure differences between the different groups, considering statistically significant when p<0.05.
